# Sickle cell trait is associated with controlled levels of haem and mild proinflammatory response during acute malaria infection

**DOI:** 10.1111/cei.12936

**Published:** 2017-02-28

**Authors:** T. W. Ademolue, O. K. Amodu, G. A. Awandare

**Affiliations:** ^1^ West African Centre for Cell Biology of Infectious Pathogens, Department of Biochemistry, Cell and Molecular BiologyCollege of Basic and Applied Sciences, University of GhanaLegonGhana; ^2^ Institute of Child HealthUniversity of IbadanNigeria

**Keywords:** haem, haemoxygenase‐1, malaria, sickle cell trait

## Abstract

The controlled induction of haemoxygenase‐1 (HO‐1), an enzyme that catabolizes haem, has been shown to reduce haem, preventing pathologies associated with haem toxicity. The hemoglobin genotype HbAS confers reduced susceptibility to severe complications of malaria by a mechanism that is not well understood. Using a longitudinal approach, we investigated the effect of baseline concentrations of HO‐1 on the accumulation of haem during acute *Plasmodium falciparum* malaria in HbAS and HbAA genotypes. Plasma concentrations of haem, HO‐1 and cytokines were quantified in venous blood obtained from children (9 months–5 years of age) during malaria infection, and at convalescence (baseline levels). Parasitaemia was determined during malaria infection. In patients with the HbAA genotype, there was a significant elevation in the plasma concentration of haem (*P* = 0.002), and a consequent increased induction of HO‐1 (*P* < 0.001) during *falciparum* malaria compared with levels at convalescence. Contrary to HbAA, plasma concentration of haem did not change in the HbAS genotypical group (*P* = 0·110), and the induction of HO‐1 was reduced during malaria compared with levels at convalescence (*P* = 0·006). Higher plasma levels of haem were observed in HbAS compared with HbAA at convalescence (*P* = 0·010), but this difference did not affect the levels of HO‐1 within each genotype (*P* = 0·450). Relatively milder proinflammatory responses were observed in HbAS children during malaria infection compared to HbAA children. Our findings suggest that a mechanism of reduced susceptibility to severe malaria pathologies by the HbAS genotype may involve the control of haem, leading to controlled levels of HO‐1 and milder proinflammatory responses during acute malaria.

## Introduction

The clinical outcome of most infectious diseases, including malaria, is mediated by complex host and pathogen factors 1. Host factors include the level of host immunity (resistance) against the circulating strain of parasite and some intrinsic mechanisms that limit pathology without necessarily interfering with pathogen burden 2.

The *Plasmodium* parasites have long co‐existed, and co‐evolved, with the human host. This relationship has exerted extraordinary evolutionary pressure on the human genome. Consequently, humans have selected multiple genetic polymorphisms that provide intrinsic protection against severe malaria complications 3, 4, 5, 6. The heterozygous sickle haemoglobin genotype (HbAS) is the best‐characterized human genetic polymorphism associated with malaria. The HbS allele has increased in frequency within the African population, hitting a prevalence of 25–30% in some African populations 7. The high prevalence of HbS allele in sub‐Saharan Africa and some other tropical areas is presumed to be due to the protection against severe malaria afforded to heterozygotes 8, 9, 10.

Associations between the HbAS genotype and protection against severe forms of malaria disease have been established 8, 11. One mechanism postulates that the HbAS genotype causes reduced parasite burdens 12. However, several other studies have suggested that the HbAS genotype limits pathological conditions such as cyto‐adherance 13 and immune‐modulatory imbalance 14, without necessarily interfering or limiting parasite burden 15, 16, 17. The evidence to date demonstrates that children with the HbAS genotype contract malaria, but they have a decreased incidence of the two common forms of severe malaria: cerebral malaria and severe malarial anaemia 11. In addition, lower mortalities from malaria are associated with this genotype 11. However, the exact molecular mechanism that explains this reduced susceptibility to severe complications of malaria and mortality compared with wild‐type genotype (HbAA) remains unclear.

Free haem has been shown to aggravate oxidative stress; thus, inducing an excessive proinflammatory state which has been associated with several pathologies, such as acute chest syndrome and lung inflammation 18, 19. The erythrocytic cycle of *P. falciparum* invasion, replication and schizont rupture leads to haemolysis and the release of haem. Thus, the accumulation of haem during malaria is expected to be involved in severe malaria complications. Indeed, reports have implicated free haem in the pathogenesis of cerebral and non‐cerebral complications of malaria in well‐defined murine models 17, 20.

Recently, the induction of haemoxygenase‐1 (HO‐1) was shown to prevent the onset of experimental cerebral malaria and mortality in *P. berghei* ANKA‐infected mice with the HbS allele 16. Ferreira and colleagues showed elegantly that the mechanism that prevented the HbAS genotype from the onset of experimental cerebral malaria in mice was independent of the abrogation of parasite burden, but via a mechanism that involves the accelerated breakdown of haem by HO‐1 16. However, the pathophysiology of *P. berghei* malaria is quite different from that of *P. falciparum*. Therefore, it is of interest to know whether this interesting phenomenon observed in murine malaria is relevant and observable in human malaria.

Based on the available evidence, we investigated the effect of the levels of haem observed in children with the HbAS and HbAA genotype on the plasma concentration of HO‐1, and the association between plasma concentration of HO‐1 and the availability of haem during malaria infection in humans. We also investigated how levels of free haem relate to the inflammatory response in the context of malaria infection.

We present data that suggest that the HbAS genotype is associated with a better control of haem accumulation during *falciparum* malaria than the HbAA genotype. The controlled level of haem during acute malaria infection was associated with a milder proinflammatory response in children with the HbAS genotype compared to the HbAA genotype. However, HO‐1 did not appear to be associated with reducing haem accumulation.

## Materials and methods

### Study site

The study was conducted at Oni Memorial Hospital, a child out‐patient clinic in Ibadan, South‐Western Nigeria. The area is a holo‐endemic region characterized by year‐round malaria transmission, with seasonal peaks between June–July and November–December 9. In the study site, malaria presents as a range of sequelae ranging from mild presentation (uncomplicated malaria) to severe life‐threatening complications, such as severe malarial anaemia and cerebral malaria 7, 9. The sampling period was January–March 2016, a lower transmission season characterized by lower malaria incidence. Almost all the children recruited into this study (90%) were from the Yoruba ethnic group, the major ethnic group in the study area 7, 9.

### Study design and study participants

**Figure 1:**
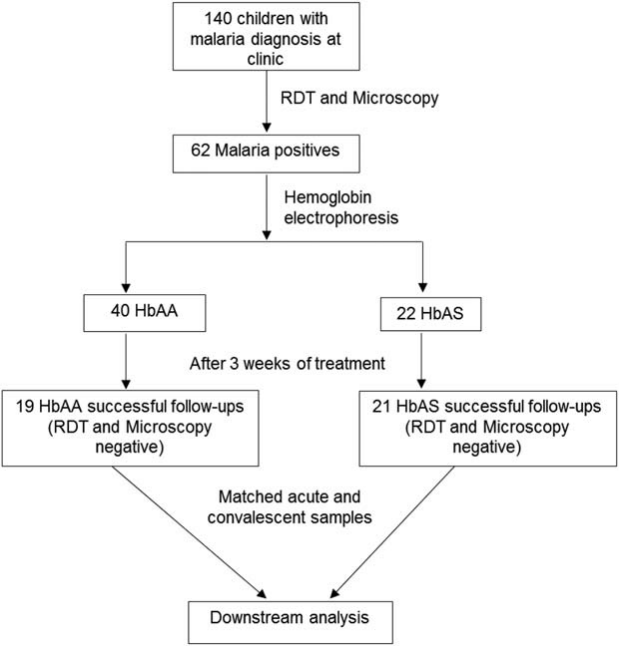
Flowchart describing patient recruitment process. One hundred and forty patients were referred for malaria tests, while 62 patients tested positive to both malaria tests, rapid diagnostic test (RDT) and microscopy. Forty‐two patients had the HbAA genotype while 20 patients were of the HbAS genotype. Forty patients (19 HbAS and 21 HbAA) were followed‐up successfully for 3 weeks, and resampled after testing negative to both malaria tests. Matched acute malaria and convalescence samples were used in downstream experiments, which included quantification of haem, haemoxygenase‐1 (HO‐1) and cytokine levels.

We adopted a longitudinal approach, which involved sampling at two time‐points: at presentation with acute malaria and at convalescent phase of infection (3 weeks later). The participants comprised 140 unrelated children (between the ages of 9 and 60 months) referred for malaria tests by the attending physician. Children who tested positive to malaria rapid diagnostic tests and were confirmed by microscopy were recruited for further laboratory analyses (*n* = 62, HbAA = 40, HbAS = 22) (Fig. 1). All patients who tested positive to both malaria tests presented signs of uncomplicated malaria, exhibiting fever or a history of fever in the past 48 h, without World Health Organization (WHO) warning signs for severe malaria 21. The children were treated with artemether/lumefantrine (Coartem), administered orally. Three weeks post‐first visit, 40 children were resampled (HbAA = 19, HbAS = 21) after parasite clearance was confirmed by rapid diagnostic test (RDT) and microscopy. Based on the negative RDT and microscopy results from the 40 patients who were followed‐up successfully, we can infer that treatment was 100% successful. However, we could not assess treatment success from the rest of the 22 children who were treated but were lost to follow‐up. This sample, taken in healthy children at convalescence, served as an internal control for each patient, and was assumed to represent predisease baseline (Fig. 1). At both sampling points (i.e. before delivery of anti‐malaria and 3 weeks post‐treatment), venous blood (2–3 ml) was drawn into sterile ethylenediamine tetraacetic acid (EDTA) tubes and transported to the clinical laboratory at the Institute of Child Health, College of Medicine, University of Ibadan. The blood samples were centrifuged and the plasmas were aliquoted into Eppendorf tubes and stored frozen at −80^°^C until further analyses. Ethical approval was obtained from the joint University of Ibadan–University College Hospital, Ibadan Ethical Committee, and from the Oyo State Health board. In addition, participation in this study was voluntary, with written informed consent obtained from the parent/guardian.

### Quantification of parasitaemia

Parasitaemia was confirmed and estimated by the microscopic examination of Giemsa‐stained thick blood films for erythrocytic stages of *P. falciparum*. The entire smear was first screened at a low magnification (×40 objective lens) to detect the best fields with even distribution of white blood cells (WBC) (10–20 WBC/field) 22. All Giemsa‐stained thick smears were viewed independently by two experienced microscopists. The results were compared, and any sample with discordant results was examined by a third experienced microscopist. Parasite densities were recorded as a ratio of parasites to WBCs in thick blood films. An assumed WBC count of 8000/μl of blood 22 was used in the calculation of parasite densities.

### Haemoglobin genotyping

Laboratory characterization of the haemoglobin (Hb) types was carried out by cellulose acetate electrophoresis at pH 8·9, as described by Chanarin 23 and adopted by Agarwal 24. The haemoglobin electrophoretic pattern of each sample haemolysate was used to assign the respective haemoglobin genotype.

### Quantification of total haem and HO‐1

Total haem (protein‐bound and free haem) in plasma was quantified by a chromogenic assay, as described previously 20, 25 using the QuantiChromHaem Assay Kit (BioAssay Systems, Hayward, CA, USA). The optical density (OD) at 400 nm wavelength of each sample was determined by a microplate reader (Bio‐Tek Services Inc., Richmond, VA, USA). The total haem concentration of each sample was calculated as described by the manufacturer. Levels of soluble HO‐1 were determined in the same plasma samples (diluted to 1 : 50) 26 by enzyme‐linked immunosorbent assay (ELISA) in duplicate wells of a precoated (with HO‐1 specific monoclonal antibodies) 96‐well strip plate using the human HO‐1 ELISA Kit (LifeSpan BioSciences Inc., Seattle, WA, USA). In addition, extensive genetic variations, which might confound levels of haem and HO‐1, were eliminated, as measurements were made in matched samples from the same individual taken at convalescence and during malaria infection.

### Cytokine profiling

Plasma concentrations of cytokines were measured in a multiplex assay using the highly sensitive xMAP technology (Luminex Corporation, Austin, TX, USA), which allows the quantification of several biological analytes in a 96‐well format. Using this method, six proinflammatory and four anti‐inflammatory cytokines were measured in duplicate wells for each sample. The proinflammatory cytokines were tumour necrosis factor (TNF)‐α, interferon (IFN)‐γ, interleukin (IL)−1β, IL‐2, IL‐6 and IL‐12 (p70), while IL‐4, IL‐7, IL‐10 and IL‐13 were the anti‐inflammatory cytokines quantified. The levels of each cytokine were recorded after background subtraction.

### Statistical analyses

Statistical analyses were performed using GraphPrism version 6.01 (GraphPad Software, Inc., San Diego, CA, USA). Parasite densities were compared between genotypes by Mann–Whitney *U*‐test. Differences between plasma levels of cytokines, HO‐1 and haem were compared within and between genotypes using either two‐sided Student's *t*‐test or Mann–Whitney *U*‐test. For association analysis, Pearson's correlation was used to investigate the relationship between parasite density and plasma concentration of haem within each genotype.

## Results

### Patients' characteristics and parasite density between genotypes during malaria infection

**Figure 2:**
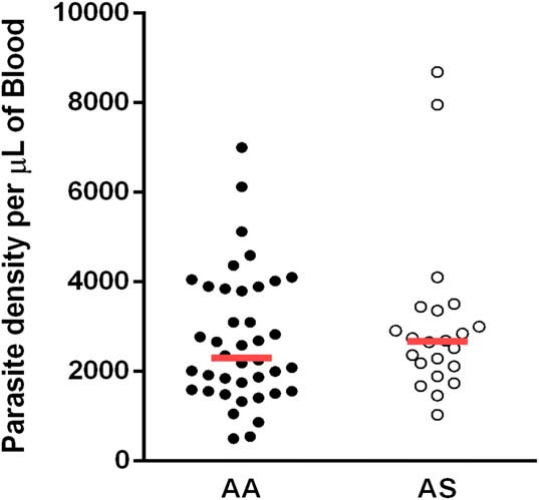
Comparison of parasite densities between both genotypic groups. Parasitaemia was determined by microscopy from Giemsa‐stained thick smears. Data are presented as scatter‐plots. The red line across indicates median value (*P* = 0·467, Mann–Whitney *U*‐test). [Colour figure can be viewed at wileyonlinelibrary.com]

One hundred and forty children who were referred for malaria tests by the attending physician were recruited into the study. The proportions of the various haemoglobin genotypes were HbAA = 66%, HbAS = 25%, HbAC = 5%, HbSC < 1% and HbSS < 4% in the entire population. Samples from children with the HbSS, HbAC and HbSC genotypes were excluded from other analyses due to very low numbers. From the total recruited children, 62 tested positive to malaria including 26 (40%) males and 36 (60%) females, with a mean age of 36 (range = 9–96) months. There were no significant differences in age (*P* = 0·200) and gender (*P* = 0·740) when they were stratified based on haemoglobin genotypes (i.e. HbAS and HbAA). Forty children with either HbAS or HbAA genotype were found to be parasite‐free by microscopy 3 weeks later (HbAS, *n* = 21; HbAA, *n* = 19). Among children with malaria, parasite density did not differ significantly between genotypes (*P* = 0·467) (Fig. 2).

### Comparison of haem levels during acute malaria infection in both genotypes

**Figure 3:**
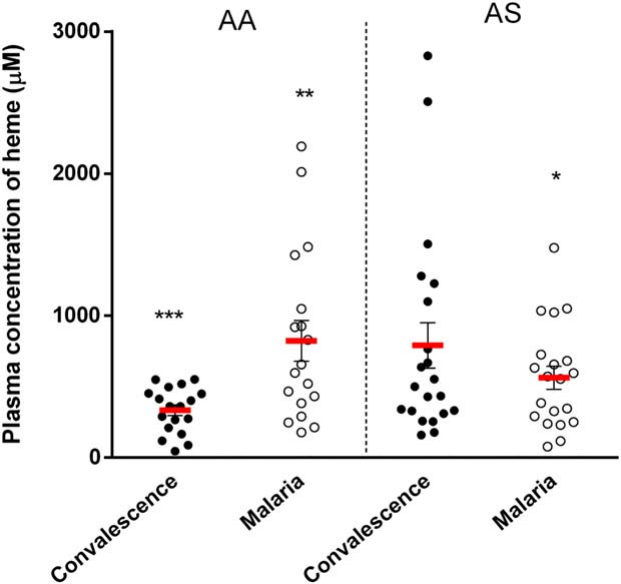
Comparison of levels of extracellular haem at baseline and during acute malaria between both genotypical groups. Plasma concentration of haem was quantified using a chromogenic assay in duplicate wells for each sample. Data are presented as scatter‐plots. Red line across indicates the mean, while error bars indicate standard error of the mean. Patients with the HbAS genotype had higher concentration of haem at the convalescent phase of malaria (*P = 0·010 for AA *versus* AS at convalescence). Patients with the HbAA genotype had higher levels during malaria infection (** *P* = 0·026 for AA *versus* AS during acute malaria). Plasma concentration of haem increased within patients with the HbAA genotype (***P = 0·002 for convalescence *versus* malaria in AA) while no significant change was observed in patients with the HbAS genotype, *P* = 0·110 (Student's *t*‐test). [Colour figure can be viewed at wileyonlinelibrary.com]

To investigate the effect of haemoglobin genotype on the accumulation of haem during malaria infection, levels of haem were measured in matched samples, with samples taken at convalescence considered as pre‐disease baseline controls. During malaria infection, patients with the HbAA genotype had higher levels of haem compared to patients in the HbAS group (*P* = 0.026) (Fig. 3). In addition, malaria infection caused a significant increase in haem levels, when levels of haem at convalescence were compared with levels during malaria infection within the HbAA group (*P* = 0·002) (Fig. 3), whereas no significant difference in haem levels were observed within the HbAS group (*P* = 0·110) (Fig. 3). Interestingly, when levels of haem at convalescence were compared between genotypes, patients with the HbAS genotype had significantly higher levels of circulating extracellular haem compared to patients with the HbAA genotype (*P* = 0·010) (Fig. 3). Taken together, these data show that acute malaria infection does not cause a marked increase in haem levels in patients with the HbAS genotype, and suggests a better control of haem.

### Parasite density does not correlate with plasma concentration of haem during malaria infection in patients with the HbAS genotype

**Figure 4:**
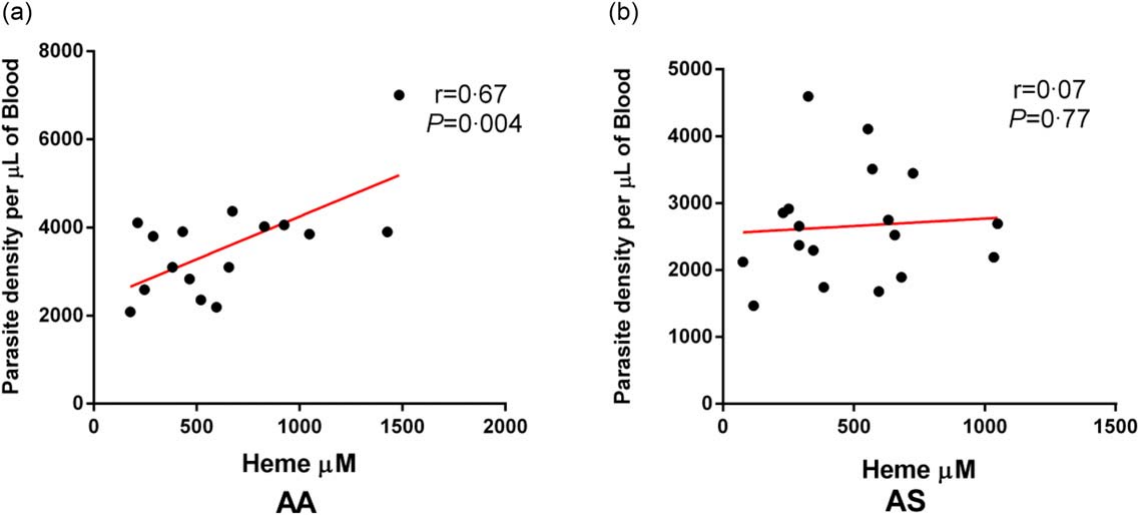
Correlation of parasitaemia with plasma concentration of haem during malaria infection within each genotype. Parasite density as quantified by microscopy (a) correlated positively with plasma concentration of haem during acute malaria infection in non‐carriers alone (HbAA, Pearson's correlation analysis *r* = 0·67, *P* = 0·004), (b) HbAS, Pearson's correlation analysis *r* = 0·07, *P* = 0·770. [Colour figure can be viewed at wileyonlinelibrary.com]

The egress of matured merozoites from schizonts is accompanied by the release of undigested haemoglobin and unpolymerized haem 27, 28. Consequently, parasitaemia is expected to contribute to the plasma concentration of extracellular haem during malaria infection. Based on this hypothesis, we investigated the association of parasite density with plasma levels of haem during malaria infection in both haemoglobin genotypes. Pearson's correlation analysis revealed a significant positive correlation between parasite density and plasma levels of haem in patients with HbAA genotype (*r* = 0·67, *P* = 0·004) (Fig. 4a) only, but not in patients with the HbAS genotype (*r* = 0·07, *P* = 0·770) (Fig. 4b). These data suggest a linear association between parasitaemia and haem levels within the HbAA group only, and confirms that patients with the HbAS genotype might be able to control haem levels more effectively during malaria infection.

### Baseline concentration of HO‐1 is not associated directly with the control of haem in patients with the HbAS genotype during malaria infection

**Figure 5:**
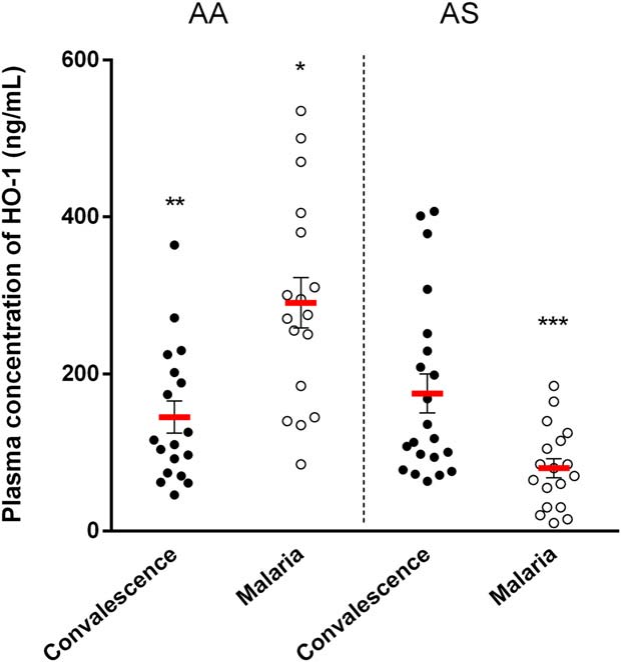
Comparison of plasma concentration of haemoxygenase‐1 (HO‐1)at baseline and during malaria infection between both genotypical groups. Plasma concentration of HO‐1 was measured by enzyme‐linked immunosorbent assay (ELISA) in duplicate wells for each sample. Data are presented as scatter‐plots. Red line across indicates the mean, while error bars indicate standard error of the mean. Plasma concentration of HO‐1 did not differ at baseline (convalescence) between the two genotypes (*P* = 0·750 for AA *versus* AS at convalescence). Patients with the HbAA genotype had higher plasma concentration of HO‐1 compared with patients with HbAS genotype during malaria infection (*P < 0·001 for AA *versus* AS during acute malaria). Plasma concentration of HO‐1 increased significantly in patients with the HbAA genotype during malaria infection (**P < 0·001 for convalescence *versus* malaria), while it decreased in patients with the HbAS genotype during malaria infection (***P = 0·006 for convalescence *versus* malaria) (Student's *t*‐test). [Colour figure can be viewed at wileyonlinelibrary.com]

Increased induction of HO‐1 has been shown to control levels of haem 16; we consequently investigated whether higher baseline levels would be associated with the better control of haem that was observed in patients with the HbAS genotype during acute malaria. Levels of HO‐1 measured at convalescence were taken as predisease baseline levels. The concentration of circulating HO‐1 at convalescence did not differ significantly between the genotypes as quantified by ELISA (*P* = 0·750) (Fig. 5); however, levels of HO‐1 were significantly higher in the HbAA group compared with the HbAS group during malaria infection (*P* < 0·001) (Fig. 5). A significant increase in the induction of HO‐1 marked by an increased plasma concentration was observed in patients with the HbAA genotype during acute malaria compared with levels at convalescence (*P* < 0·001) (Fig. 5). In contrast, there was a significant decrease in the plasma concentration of HO‐1 in patients with the HbAS genotype during malaria infection compared with levels at convalescence (*P* = 0·006) (Fig. 5). As levels of HO‐1 did not differ significantly between genotypes at convalescence, we can infer that baseline levels of HO‐1 may not play a direct role in controlling haem levels during malaria infection in patients with the HbAS genotype.

### Milder proinflammatory response during malaria infection in patients with the HbAS genotype

**Figure 6:**
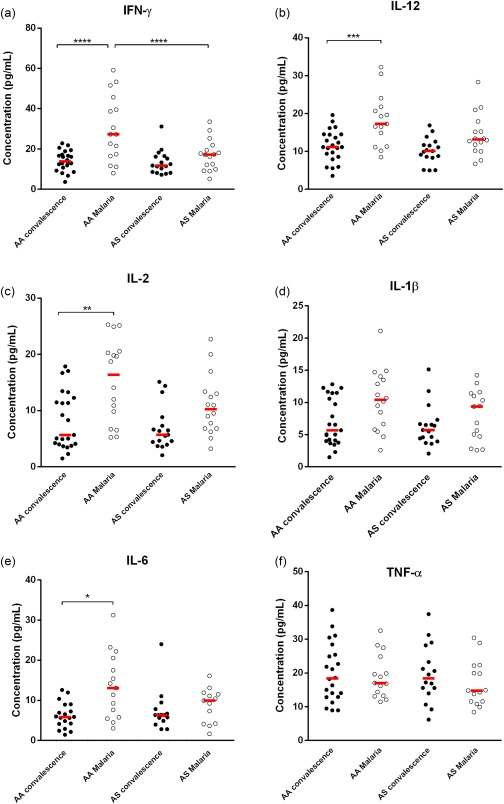
Proinflammatory cytokine profile within each genotype during malaria infection. Data are presented as scatter‐plots. The line across represents the median value (*P < 0·050; **P < 0·010; ***P < 0·001; ****P < 0·0001; Mann–Whitney *U*‐test). [Colour figure can be viewed at wileyonlinelibrary.com]

An excessive proinflammatory response has been implicated as a contributing factor to severe malaria pathologies 14, 29. As extracellular haem can activate immune cells and cause the excessive secretion of proinflammatory cytokines 30, 31, we investigated whether higher levels of haem that were observed in patients with the HbAA genotype were associated with an increased inflammatory response during malaria infection. As quantified by a multiplex assay, no significant increase in the plasma concentrations of IFN‐γ, IL‐12, IL‐2, IL‐1β and IL‐6 was observed in patients with the HbAS genotype during malaria infection compared with levels at convalescence (*P* = 0·620, *P* = 0·090, *P* = 0·150, *P* = 0·890 and *P* = 0·450, respectively) (Fig. 6a–e). However, levels of these proinflammatory cytokines increased significantly during malaria infection in patients with the HbAA genotype (*P <* 0·001*, P* < 0·001*, P* = 0·001, *P* = 0·045 and *P* < 0·001, respectively) (Fig. 6a–e). In addition, IFN‐γ levels were elevated significantly during malaria infection in patients with the HbAA genotype compared with the HbAS genotype (*P* = 0·008) (Fig. 6a). Levels of TNF‐α did not increase significantly within each genotype during malaria infection compared with baseline concentration (HbAA: *P* = 0·990; HbAS *P* = 0·740) (Fig. 6f). Taken together, this result suggests that children with the HbAS genotype are better able to control the proinflammatory response during acute malaria, presumably because they are better able to control levels of haem.

### Similar patterns of anti‐inflammatory response during malaria infection in HbAA and HbAS genotypes

**Figure 7:**
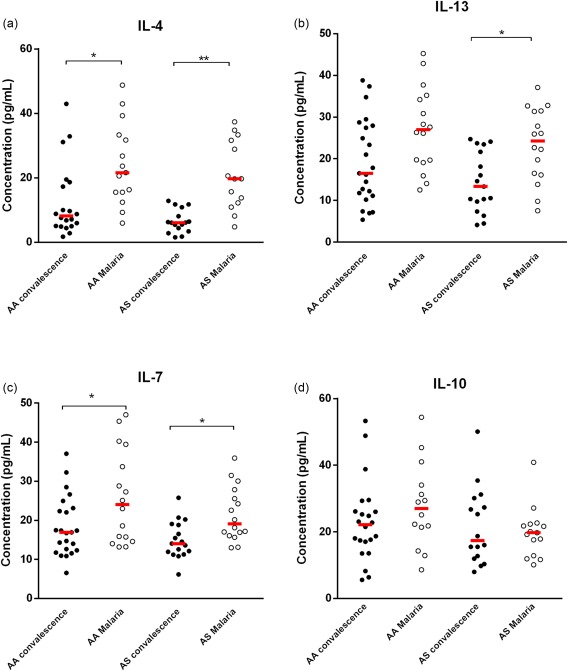
Anti‐inflammatory cytokine profile within each genotype during malaria infection. Data are presented as scatter‐plots. The line across represents the median value (*P < 0·050; **P < 0·010, ***P < 0·001; ****P < 0·000; Mann–Whitney *U*‐test). [Colour figure can be viewed at wileyonlinelibrary.com]

Plasma concentrations of four anti‐inflammatory cytokines (IL‐4, IL‐7, IL‐13 and IL‐10) were also quantified in the two groups at convalescence and during malaria infection. Levels observed during malaria infection were compared with levels at convalescence. Similar patterns of anti‐inflammatory response were observed within each genotype. For IL‐4 and IL‐7, plasma concentrations increased significantly during malaria infection within both genotypical groups (HbAA IL‐4 *P* = 0·010, IL‐7 *P* = 0·010; HbAS IL‐4 *P* < 0·001, IL‐7 *P* = 0·004) (Fig. 7a, b). Levels of IL‐13 also increased significantly during malaria infection in both genotypical groups (HbAS, *P* = 0·030 and HbAA, *P* = 0·050); however, the increase was at borderline significance in the HbAA group. Plasma concentrations of IL‐10 did not differ significantly during malaria infection compared with levels at convalescence in both haemoglobin genotypes (HbAA, *P* > 0·900; HbAS, *P* > 0·900) (Fig. 7d).

## Discussion

We adopted a longitudinal approach to investigate the hypothesis that chronic haemolysis in patients with the HbAS genotype leads to increased levels of haem, which consequently induces higher chronic levels of HO‐1. High chronic levels of HO‐1 would, in turn, cause the accelerated breakdown of haem released during parasite egress, thus preventing excessive inflammation. Studies in murine models have demonstrated that the accelerated breakdown of haem by the increased induction of HO‐1 prevents the onset of experimental cerebral malaria in mice 16, 17. Contrary to reports that have implicated the reduction of parasitaemia as a cause of the reduced susceptibility to severe complications of malaria in patients with the HbAS genotype 3, 12, 32, we found no significant difference in parasite densities between patients with the HbAS genotype and patients with the HbAA genotype during acute malaria infection. Our cohort of children does not have severe forms of malaria; therefore, we cannot rule out the possibility of a difference in parasitaemia between the genotypical groups if they have severe malaria. In addition, this study was constrained by low sample size, therefore a larger sample size should be considered in subsequent studies. Nevertheless, it is possible that the HbAS genotype mediates reduced susceptibility to severe complications of malaria independently of the reduction of parasite burden, as shown in previous reports 16, 17, 20, 26, and as we have observed from our population during acute malaria infection.

Although parasitaemia did not differ between haemoglobin genotypes, plasma levels of haem were lower in patients with the HbAS genotype compared with patients with the HbAA genotype during malaria infection, suggesting that carriers of this trait might be better able to control haem during malaria infection. Further analysis revealed that parasite density correlated positively with plasma levels of haem during malaria infection in patients with the HbAA genotype alone. The correlation analysis was confirmed further from the observation of higher plasma concentrations of haem during acute malaria infection compared with levels at convalescence in the HbAA genotype, while plasma concentrations of haem did not change in patients with the HbAS genotype during acute malaria infection compared with levels at convalescence. The increase in plasma levels of haem during malaria infection in patients with the HbAA genotype can be attributed to haemolysis driven by the lysis of erythrocytes during merozoite egress 5, 6, 28. However, the non‐significant change in the concentration of haem during acute malaria in patients with the HbAS genotype suggests that these individuals may have a more efficient mechanism for the breakdown of haem.

Evidence from previous studies have shown that haem, the substrate of HO‐1, is also its major inducer 15, 16, 17, 20, 33, and when cells are exposed to haem there is an increased expression of HO‐1 to catabolize haem and prevent its cytotoxicity 33. As patients with the HbAS genotype have higher levels of haem compared with HbAA genotype at convalescence, we investigated the possibility of a corresponding induction of HO‐1 in both genotypes. Our findings demonstrate the first evaluation of baseline levels of HO‐1 in children with different haemoglobin genotypes. A previous study demonstrated that malaria‐induced soluble mediators returned to normal levels 1 week after treatment 34; therefore, levels of HO‐1 measured 3 weeks post‐treatment (at convalescence) is a valid approximation of baseline levels. We observed that the difference in the concentrations of haem between genotypes at convalescence did not affect levels of HO‐1 significantly, although patients with the HbAS genotype had slightly higher levels at convalescence. Previous studies have shown that extracellular haem is scavenged by haemopexin 35, and when haemopexin is saturated, excess free haemin is adsorbed to albumin 15. These proteins represent different levels of haem scavangers. When the scavenging capacity of haemopexin and albumin is exhausted, then HO‐1 presents a final defence mechanism that avoids the accumulation of free haem in plasma 36, 37. Therefore, the observation that levels of HO‐1 at convalescence were not affected significantly by levels of haem at convalescence within the HbAS group suggest that these described first layers of haem scavengers may be regulating circulating levels of haem effectively with no significant effect on HO‐1 levels. In summary, the baseline concentration of HO‐1 may not play a (direct) role in the better control of haem that we observed in the HbAS group during malaria infection.

The increased induction of HO‐1 in HbAA patients during acute malaria poses a danger. Haem‐induced HO‐1 above a certain threshold may be part of the causal pathway leading to severe diseases 25. Suttner and colleagues demonstrated how haem can mediate oxidative injury by increasing HO‐1 expression 38. They observed that ferrous iron, a by‐product of haem breakdown, accumulated in excessively high HO‐1‐expressing cells 38. Therefore, HO‐1 at very high levels may potentiate, rather than attenuate, cellular toxicity from reactive oxygen species (ROS). Data presented here show that patients with the HbAA genotype indeed have a higher plasma concentration of HO‐1 during malaria infection than patients with the HbAS genotype, suggesting a higher predisposition of the HbAA group to oxidative stress.

Free haem can activate immune cells causing immune‐modulatory imbalance, which can lead to the secretion of proinflammatory cytokines in excess 14, 29. Measurement of mediators of inflammation revealed that proinflammatory cytokines (including IL‐12, IFN‐α, IL‐1β, IL‐6 and IL‐2) increased significantly in patients with the HbAA genotype during malaria infection compared with baseline levels, while no significant elevation in plasma concentrations of these proinflammatory cytokines were observed in patients with the HbAS genotype. We also quantified anti‐inflammatory cytokines, as a balance of pro‐ and anti‐inflammatory cytokines is necessary to control inflammatory responses 29. Unlike the proinflammatory cytokines, patterns of anti‐inflammatory cytokines were mostly similar in the AA and AS groups. We examined correlations of haem levels with cytokine levels during malaria infection within each genotype; however, we found no significant linear associations between levels of haem and cytokine levels (data not shown), indicating the complexity of the relationship between haem levels and cytokine regulation.

Higher levels of proinflammatory cytokines have been shown to cause dyserythropoiesis 30, which can lead to severe malarial anaemia. In addition, proinflammatory cytokines cause an increased expression of adhesion molecules on vascular endothelial cells, which promotes sequestration of infected erythrocytes. Sequestration of infected erythrocytes in the microvasculature of the brain can lead to cerebral malaria 13, 14, 26, 39. Plasma concentrations of IFN‐γ were also significantly higher in patients with the HbAA genotype compared with patients with the HbAS genotype during acute malaria infection. IFN‐γ is a potent mediator of proinflammation and plays essential roles in protective immunity against blood‐stage *Plasmodium* infection. However, after *Plasmodium* infection, increased IFN‐γ production by various types of cells, particularly CD4^+^ T cells, is involved not only in protective immunity, but also in immune pathology 40, 41, 42. These results suggest that a higher plasma concentration of haem might influence a higher secretion of proinflammatory cytokines, particularly IFN‐γ, in patients with the HbAA genotype, which may predispose them to excessive inflammation that has been associated with severe malaria pathologies 14. Taken together, these results indicate an increased induction of the type 1 proinflammatory response in patients with the HbAA genotype compared with patients with the HbAS genotype.

Observations from this study provide insights into a possible mechanism of reduced susceptibility to severe complications of malaria in patients with the HbAS genotype. We showed that patients with the HbAS genotype are able to control haem during acute malaria infection. The ability to control haem effectively led to milder proinflammatory responses in patients with the HbAS genotype compared to the HbAA genotype. The effective control of haem and proinflammatory cytokines during acute malaria may explain the reduced susceptibility of children with the HbAS genotype to severe complications of malaria.

## Author contributions

T. W. A. and G. A. A. designed the study and wrote the paper. G. A. A. and O. K. A. co‐supervised the experiments and edited the manuscript.

## Disclosure

The authors declare no disclosures.

## References

[1] Mohandas N , An X. Malaria and human red blood cells. Med Microbiol Immunol 2012; 201:593–8. 22965173 10.1007/s00430-012-0272-z PMC3699179

[2] Safeukui‐Noubissi I , Ranque S , Poudiougou B *et al* Risk factors for severe malaria in Bamako, Mali: a matched case–control study. Microbes Infect 2004; 6:572–8. 15158191 10.1016/j.micinf.2004.02.007

[3] Allison AC. Genetic factors in resistance to malaria. Ann NY Acad Sci 1961; 91:710–29. 13682588 10.1111/j.1749-6632.1961.tb31102.x

[4] Mackinnon MJ , Mwangi TW , Snow RW , Marsh K , Williams TN. Heritability of malaria in Africa. PLOS Med 2005; 2:e340. 16259530 10.1371/journal.pmed.0020340 PMC1277928

[5] Williams TN. Human red blood cell polymorphisms and malaria. Curr Opin Microbiol 2006; 9:388–94.] 16815736 10.1016/j.mib.2006.06.009

[6] Taylor SM , Fairhurst RM. Malaria parasites and red cell variants: when a house is not a home. Curr Opin Hematol 2014; 21:193–200. 24675047 10.1097/MOH.0000000000000039 PMC4083250

[7] Olaniyan SA , Amodu OK , Bakare AA , Omotade OO , Rockett KA , Consortium M. Tumour necrosis factor alpha promoter polymorphism, Tnf− 238 is associated with severe clinical outcome of *Falciparum malaria* in Ibadan, Southwest Nigeria. Acta Tropica 2016; 161:62–7. 27178813 10.1016/j.actatropica.2016.05.006

[8] Amoako N , Asante KP , Adjei G , Awandare GA , Bimi L , Owusu‐Agyei S. Associations between red cell polymorphisms and *Plasmodium falciparum* infection in the middle belt of Ghana. PLOS ONE 2014; 9:e112868. 25470251 10.1371/journal.pone.0112868 PMC4254276

[9] Amodu OK , Olaniyan SA , Adeyemo AA , Troye‐Blomberg M , Olumese PE , Omotade OO. Association of the sickle cell trait and the ABO blood group with clinical severity of malaria in Southwest Nigeria. Acta Trop 2012; 123:72–7. 22503377 10.1016/j.actatropica.2012.03.013

[10] Piel FB , Patil AP , Howes RE *et al* Global distribution of the sickle cell gene and geographical confirmation of the malaria hypothesis. Nat Commun 2010; 1:104.] 21045822 10.1038/ncomms1104 PMC3060623

[11] Lopera‐Mesa TM , Doumbia S , Chiang S *et al* *Plasmodium falciparum* clearance rates in response to artesunate in Malian children with malaria: effect of acquired immunity. J Infect Dis 2013; 207:1655–63. 23448727 10.1093/infdis/jit082 PMC3636783

[12] Allison AC. Protection afforded by sickle‐cell trait against subtertian malarial infection. Brit Med J 1954; 1:290. 13115700 10.1136/bmj.1.4857.290 PMC2093356

[13] Beaudry JT , Krause MA , Diakite SA *et al* *Ex‐vivo* cytoadherence phenotypes of *Plasmodium falciparum* strains from Malian children with hemoglobins A, S, and C. PLOS ONE 2014; 9:e92185. 24647281 10.1371/journal.pone.0092185 PMC3960211

[14] Mackintosh CL , Beeson JG , Marsh K. Clinical features and pathogenesis of severe malaria. Trends Parasitol 2004; 20:597–603. 15522670 10.1016/j.pt.2004.09.006

[15] Ferreira A , Balla J , Jeney V , Balla G , Soares MP. A central role for free heme in the pathogenesis of severe malaria: the missing link? J Mol Med (Berl) 2008; 86:1097–111. 18641963 10.1007/s00109-008-0368-5

[16] Ferreira A , Marguti I , Bechmann I *et al* Sickle hemoglobin confers tolerance to plasmodium infection. Cell 2011; 145:398–409. 21529713 10.1016/j.cell.2011.03.049

[17] Seixas E , Gozzelino R , Chora A *et al* Heme oxygenase‐1 affords protection against noncerebral forms of severe malaria. Proc Natl Acad Sci USA 2009; 106:15837–42. 19706490 10.1073/pnas.0903419106 PMC2728109

[18] Ghosh S , Adisa OA , Chappa P *et al* Extracellular hemin crisis triggers acute chest syndrome in sickle mice. J Clin Invest 2013; 123:4809–20. 24084741 10.1172/JCI64578 PMC3809772

[19] Lam A , Vetal N , Matalon S , Aggarwal S. Role of heme in bromine‐induced lung injury. Ann NY Acad Sci 2016; 1374:105–10. 27244263 10.1111/nyas.13086 PMC4940273

[20] Pamplona A , Ferreira A , Balla J *et al* Heme oxygenase‐1 and carbon monoxide suppress the pathogenesis of experimental cerebral malaria. Nat Med 2007; 13:703–10. 17496899 10.1038/nm1586

[21] World Health Organization , Severe malaria. Trop Med Int Health 2014; 19:7–131. 25214480 10.1111/tmi.12313_2

[22] Guy M. Basic malaria microscopy. Parasitol Today 1992; 8:319.

[23] Chanarin I. Hematology: principles and procedures. J Clin Path 1984; 37:1419.

[24] Agarwal A , Guindo A , Cissoko Y *et al* Hemoglobin C associated with protection from severe malaria in the Dogon of Mali, a West African population with a low prevalence of hemoglobin S. Blood 2000; 96:2358–63. 11001883

[25] Walther M , De Caul A , Aka P *et al* Hmox1 gene promoter alleles and high HO‐1 levels are associated with severe malaria in Gambian children. PLOS Pathog 2012; 8:e1002579. 22438807 10.1371/journal.ppat.1002579 PMC3305414

[26] Beaudry JT , Fairhurst RM. Microvascular sequestration of *Plasmodium falciparum* . Blood 2011; 117:6410. 21823261 10.1182/blood-2010-09-305102 PMC3123013

[27] Goldberg DE , Slater AF , Cerami A , Henderson GB. Hemoglobin degradation in the malaria parasite *Plasmodium falciparum*: an ordered process in a unique organelle. Proc Natl Acad Sci USA 1990; 87:2931–5. 2183218 10.1073/pnas.87.8.2931 PMC53807

[28] Hebbel RP , Morgan WT , Eaton JW , Hedlund BE. Accelerated autoxidation and heme loss due to instability of sickle hemoglobin. Proc Natl Acad Sci USA 1988; 85:237–41. 3422420 10.1073/pnas.85.1.237 PMC279519

[29] Clark IA , Alleva LM , Budd AC , Cowden WB. Understanding the role of inflammatory cytokines in malaria and related diseases. Travel Med Infect Dis 2008; 6:67–81. 18342278 10.1016/j.tmaid.2007.07.002

[30] Perkins DJ , Were T , Davenport GC , Kempaiah P , Hittner JB , Ong'echa JM. Severe malarial anemia: innate immunity and pathogenesis. Int J Biol Sci 2011; 7:1427–42. 22110393 10.7150/ijbs.7.1427 PMC3221949

[31] Wagener FA , Feldman E , de Witte T , Abraham NG . Heme Induces the Expression of Adhesion Molecules Icam‐1, Vcam‐1, and E Selectin in Vascular Endothelial Cells. Proceedings of the Society for Experimental Biology and Medicine 1997; 216:456–63. 9402154 10.3181/00379727-216-44197

[32] Mangano VD , Kabore Y , Bougouma EC *et al* Novel insights into the protective role of hemoglobin S and C against *Plasmodium falciparum* parasitemia. J Inf Dis 2015; 212:626–34. 10.1093/infdis/jiv098 PMC4512610 25712976

[33] Rowe JA , Claessens A , Corrigan RA , Arman M. Adhesion of *Plasmodium falciparum*‐infected erythrocytes to human cells: molecular mechanisms and therapeutic implications. Expert Rev Mol Med 2009; 11:e16. 19467172 10.1017/S1462399409001082 PMC2878476

[34] Hviid L , Kurtzhals JA , Adabayeri V *et al* Perturbation and proinflammatory type activation of Vδ1+ Γδ T cells in African children with *Plasmodium falciparum* malaria. Infect Immun 2001; 69:3190–6. 11292740 10.1128/IAI.69.5.3190-3196.2001 PMC98276

[35] Rother RP , Bell L , Hillmen P , Gladwin MT. The clinical sequelae of intravascular hemolysis and extracellular plasma hemoglobin: a novel mechanism of human disease. JAMA 2005; 293:1653–62. 15811985 10.1001/jama.293.13.1653

[36] Gozzelino R , Jeney V , Soares MP. Mechanisms of cell protection by heme oxygenase‐1. Annu Rev Pharmacol Toxicol 2010; 50:323–54. 20055707 10.1146/annurev.pharmtox.010909.105600

[37] Philippidis P , Mason JC , Evans BJ *et al* Hemoglobin scavenger receptor cd163 mediates interleukin‐10 release and heme oxygenase‐1 synthesis: antiinflammatory monocyte–macrophage responses *in vitro*, in resolving skin blisters *in vivo*, and after cardiopulmonary bypass surgery. Circ Res 2004; 94:119–26. 14656926 10.1161/01.RES.0000109414.78907.F9

[38] Suttner DM , Dennery PA. Reversal of Ho‐1 related cytoprotection with increased expression is due to reactive iron. FASEB J 1999; 13:1800–9. 10506583 10.1096/fasebj.13.13.1800

[39] Panichakul T , Payuhakrit W , Panburana P , Wongborisuth C , Hongeng S , Udomsangpetch R. Suppression of erythroid development *in vitro* by *Plasmodium vivax* . Malar J 2012; 11:173. 22624872 10.1186/1475-2875-11-173 PMC3407695

[40] Amani V , Vigário AM , Belnoue E *et al* Involvement of Ifn‐Γ receptor‐mediated signaling in pathology and anti‐malarial immunity induced by *Plasmodium berghei* infection. Euro J Immunol 2000; 30:1646–55. 10.1002/1521-4141(200006)30:6<1646::AID-IMMU1646>3.0.CO;2-0 10898501

[41] Inoue S , Niikura M , Mineo S , Kobayashi F. Roles of Ifn‐gamma and gammadelta T cells in protective immunity against blood‐stage malaria. Front Immunol 2013; 4:258. 24009610 10.3389/fimmu.2013.00258 PMC3756480

[42] Yanez DM , Manning DD , Cooley AJ , Weidanz WP , van der Heyde HC. Participation of lymphocyte subpopulations in the pathogenesis of experimental murine cerebral malaria. J Immunol 1996; 157:1620–4. 8759747

